# Levels of Practice and Determinants of Diabetes Self-Care in Primary Health Care in Jeddah City, Saudi Arabia

**DOI:** 10.7759/cureus.8816

**Published:** 2020-06-25

**Authors:** Ali H AlQahtani, Ahmed S Alzahrani, Sami H Alzahrani, Saleh M Alqahtani, Abdullah F AlOtaibi, Adeel Ahmed Khan

**Affiliations:** 1 Public Health Administration, Ministry of Health, Jeddah, SAU; 2 Preventive Medicine, Armed Forces Hospital, Madina, SAU; 3 Family Medicine, King Abdulaziz University, Jeddah, SAU; 4 Family Medicine, Ministry of Health, Jeddah East Hospital, Jeddah, SAU; 5 Public Health Administration, Ministry of Health, Makkah, SAU; 6 Epidemiology and Public Health, Ministry of Health, Saudi Board of Preventive Medicine, Mecca, SAU

**Keywords:** type 1 diabetes, type 2 diabetes, self-management, self-care, glycemic control, uncontrolled, jeddah, saudi arabia

## Abstract

Objective

To assess the level and determinants of practice in diabetes self-management at primary health care centers (PHCCs) and to analyze the association of self-management with the level of glycemic control.

Method

A cross-sectional study was conducted among patients with type 1 and type 2 diabetes, aged ≥ 17 years, and being followed at PHCCs in Jeddah, Saudi Arabia, from December 1, 2019, to December 30, 2019. A multistage cluster sampling technique was used to select 350 participants from five PHCCs. The level of practice in self-management was assessed using the Arabic version of the Summary of Diabetes Self-care Activities (SDSCA) questionnaire. The tool was administered as a face-to-face interview, followed by the collection of sociodemographic and relevant clinical data. In addition, blood was collected to measure fasting blood glucose (FBG) and HbA1c levels. The association of the overall SDSCA score with diabetes control was analyzed using linear regression and the receiver operator characteristics (ROC) curve. Multivariate binary logistic regression was carried out to analyze independent factors of inadequate practice.

Result

The overall mean (SD) SDSCA score was 3.13 (1.13)/7. Of the five dimensions of self-care, medication adherence yielded the highest score (mean=5.39 days per week), followed by diet (2.83) and blood glucose monitoring (2.78), while footcare had the lowest level of practice (2.26). The SDSCA score showed a negative correlation with the level of HbA1c, with a correlation coefficient r-squared =0.530 and regression coefficient B=-0.648 (p <0.001). ROC curve analysis showed that optimal glycemic control was associated with SDSCA score cutoff ≥3.5 with 82.0% sensitivity and 77.0% specificity, and the model showed that 38.0% of participants had adequate practice in self-management. Inadequate practice in diabetes self-management was independently associated with age >50 years (OR=2.00 [95%CI=1.02, 3.89]), rental accommodation (OR=0.42 [95%CI=0.23, 0.76]), independent job (OR=3.98 [95%CI=1.66, 9.57]), and longer duration of diabetes (≥8 years) (OR=4.25 [95%CI=1.82, 9.92]).

Conclusion

There are low levels of diabetes self-management among patients being followed at Jeddah PHCCs. This is associated with suboptimal glycemic control among the majority of the patients, indicating the importance of self-management to improve diabetes control. Patient health literacy and education for self-management should be considered the standard of care for diabetic patients in all PHCCs, with specific attention to subcategories of patients with the lowest levels of practice in self-management such as those with a longer duration of diabetes and the elderly.

## Introduction

Approximately half of the patients with diabetes are reported to have suboptimal or poor glycemic control, both at the national level and the global level [1-2]. Uncontrolled diabetes represents the major factor for diabetes-related morbidity. Notably, cardiovascular complications increase the number of hospitalizations and related health expenditures [3-5]. The level of hemoglobin A1c (HbA1c) is linearly associated with coronary heart disease (CHD) hospitalization. Uncontrolled diabetes was associated with 13.6% all-cause mortality, 17.9% for CHD, and 2.7% for stroke hospitalization [6]. A local study estimated that each unit increase in HbA1c level is independently associated with a 40% and 11% increase in the odds of microvascular and macrovascular complications, respectively [7].

Over the last two decades, self-management has become an integral part of management in patients with diabetes in combination with pharmacological treatments. It consists of empowering patients to perform a set of activities to achieve target lifestyle and behavioral standards in different dimensions such as diet, exercise, and blood glucose monitoring [8-10]. This strategy demonstrated high efficacy in improving diabetes control and is increasingly recommended as a standard of care in diabetes [2,9,11].

Consequently, increasing attention is given to diabetes self-management, with several pieces of research carried out locally, showing inadequate levels of practice in various dimensions of self-care [12-14]. Such observations prompted physicians’ and researchers’ efforts to promote patient education for self-care [15-16] and to propose technology-based solutions such as usage of smartphone applications and gamification of self-care behavior to alleviate further obstacles and produce promising results in the Kingdom [17-18]. On the other hand, national data remain scarce, stressing the need for the continuous monitoring of self-management among patients with diabetes.

The present study was conducted to provide further insights into the issue in the primary health care centers (PHCCs) of Jeddah, Saudi Arabia. We evaluated the level of practice in diabetes self-management and its association with diabetes control and explored the sociodemographic and clinical factors associated with poor practice in self-management.

## Materials and methods

Design and setting

A cross-sectional study was conducted in PHCCs in Jeddah, Saudi Arabia, from December 1, 2019, to December 30, 2019. Being the second-largest city of Saudi Arabia, Jeddah comprises 52 PHCCs, which are distributed in five health sectors supervised by the Ministry of Health (MoH) Directorate of Health Affairs.

Population

The study included Arabic-speaking male and female patients with type 1 and type 2 diabetes, aged ≥17 years, who were registered at the Chronic Diseases Clinics and attended the clinics during the study period. Patients who had a mental disability or any condition affecting their communication or decisions, those with a physical disability affecting self-care activities, and pregnant and lactating women were excluded from the study.

Sampling 

By considering a margin error of 0.05, a 95% confidence interval (95% CI), with a statistical power of 80%, the target sample size was calculated to detect a 70% rate of non-adherence to self-care, among an estimated total population of 30,000 patients with diabetes in Jeddah [19-20]. Using the formula n = (Z2 x P(1 / P))/e2, where: Z = value from standard normal distribution corresponding to desired confidence level (Z=1.96 for 95% CI), P is expected true proportion and e is desired precision (half desired CI width). The calculated sample size was 320, which was increased to 350 to compensate for the 10% nonrespondents or incomplete participation.

A multistage cluster sampling technique was used to select one PHCC from each health sector (cluster) using a simple random technique. Systematic random sampling was used to include 70 eligible patients among those attending the Chronic Diseases Clinics in each participating center, to reach the target sample size.

Tools and data collection procedure

The level of practice in self-management was assessed using the Summary of Diabetes Self-care Activities (SDSCA) questionnaire, which is a valid and reliable multidimensional scale for measuring diabetes self-management. The tool explores the weekly frequency (number of days per week) of practice in five self-management dimensions, including diet, exercise, blood glucose monitoring, footcare, and treatment adherence [21-22]. The SDSCA uses an 8-point Likert-type scale (0-7), which represents the number of days per week when the given self-care activity was performed. Scores are calculated separately for each item and the level of adherence is indicated by the mean score for each dimension; however, the scale does not categorize the participant as adherent or non-adherent. The validated Arabic version of the SDSCA (test-retest, r=0.912; Cronbach's alpha=0.76) was used in this study [23-24].

The tool was administered as a face-to-face interview, which was completed with a semi-structured form collecting the participants' sociodemographic data (age, gender, marital status, etc.) and diabetes-related clinical factors, including diabetes duration (<8 years vs ≥8 years), type of diabetes, treatment regimen (oral antidiabetic agents (OADs), insulin, or combined insulin + OADs), presence of diabetes complications (retinopathy, nephropathy, cardiovascular complications), comorbidities (hypertension and dyslipidemia), smoking status, and the use of herbal and traditional medicine for diabetes.

Blood tests

On the day of the interview, all participants underwent peripheral venous blood collection to measure fasting blood glucose (FBG) using standard techniques. Additionally, the HbA1c level was measured for participants who had no recent (<3 months) measurement in their records. Glycemic control was considered optimal if HbA1c was ≤ 7% [25].

Ethical clearance

The study was conducted in accordance with international ethical standards and the protocol was reviewed and ethically approved by the Directorate of Health Affairs, MoH, Jeddah. All participants provided informed consent and were knowledgeable of their right to withdraw from the study at any time, without any impact on their care. Confidentiality and privacy protection were ensured by anonymized data collection and coded data sharing between different interventions in data processing. 

Statistical methods

Statistical analysis was performed with Statistical Package for Social Sciences version 21.0 for Windows (IBM Corp., Armonk, NY). Categorical variables are presented as frequency and percentage while continuous variables are presented as mean ± standard deviation (SD). The reliability of the SDSCA scale was analyzed by calculating Cronbach’s alpha, and the distribution of the overall SDSCA score was analyzed using the Kolmogorov-Smirnov and Shapiro-Wilk tests. The correlation of the overall SDSCA score with the HbA1c level was analyzed using linear regression with the calculation of unstandardized regression coefficient (B) and correlation coefficient (r), as well as the receiver operator characteristics (ROC) curve of optimal glycemic control as a function of SDSCA score with the calculation of area under the curve (AUC). Youden’s index was used to determine the cutoff SDSCA value that best correlates with optimal glycemic control. Thus, the level of practice in self-management was divided into adequate and inadequate, and the chi-square test was used to analyze its association with sociodemographic and clinical factors. Multivariate binary logistic regression was carried out to analyze independent factors of inadequate practice. A p-value of <0.05 was considered to reject the null hypothesis.

## Results

Participants’ socio-demographic characteristics

Seventy patients with diabetes were included from each of the five Jeddah sectors, for a total of 350 patients; 50.6% were male and the mean (SD) age was 50.96 (10.62). The majority were married (70.0%), having secondary (35.4%) or university+ (38.6%) educational level, and from middle-income households (household income 5-15K Saudi Riyal per month, 71.1%) (Table 1).

**Table 1:** Participants’ sociodemographic characteristics (N=350). Because of missing data, all frequencies do not sum up to the total.

Parameter	Category	Frequency	Percentage
Sector	North	70	20.0
	West	70	20.0
	East	70	20.0
	Center	70	20.0
	South	70	20.0
Gender	Male	177	50.6
	Female	173	49.4
Age (years)	Mean, SD	50.96	10.62
Marital Status	Married	245	70.0
	Single	51	14.6
	Divorced	32	9.1
	Widowed	22	6.3
Educational Level	Illiterate	12	3.4
	Primary	30	8.6
	Middle school	49	14.0
	Secondary	124	35.4
	University+	135	38.6
Nationality	Saudi	317	90.6
	Non-Saudi	33	9.4
Accommodation	Ownership	150	42.9
	Rental	192	54.9
	Not Specified	8	2.2
Profession	Civil	124	35.4
	Military	29	8.3
	Freelance	64	18.3
	Student	12	3.4
	Retired	82	23.4
	Unemployed	39	11.1
Household monthly income (SAR)	<5K	47	13.4
	5-10k	130	37.1
	10-15k	119	34.0
	15-20k	42	12.0
	>20k	12	3.4

Diabetes-related health information

Type 2 diabetes was the most prevalent type (92.3%); and in 56.9% of the total participants, diabetes was diagnosed less than eight years ago. Assessment of diabetes-related morbidity showed a prevalence of cardiovascular complications (17.4%), retinopathy (30.0%), and nephropathy (8.6%). Other comorbidities showed a high prevalence of hypertension (39.1%) and dyslipidemia (42.9%), and 27.7% were active smokers. The treatment regimen included oral antidiabetic agents (OADs) in majority participants (64.6%), followed by insulin alone (19.7%), and a combination of OAD-insulin (15.1%), while two patients (0.6%) were not on any pharmacological treatment. On the other hand, 24.3% declared using herbal and traditional medicine. The mean (SD) HbA1c was 7.83% (1.00) and diabetes control was optimal (≤7.0% HbA1c) in 86 participants (24.6%) (Table 2).

**Table 2:** Diabetes-related health information.

Parameter	Category	Frequency	Percentage
Diabetes duration	<8 years	199	56.9
	≥8 years	151	43.1
Diabetes type	Type 1	27	7.7
	Type 2	323	92.3
Cardiovascular complication	No	289	82.6
	Yes	61	17.4
Retinopathy	No	245	70.0
	Yes	105	30.0
Nephropathy	No	316	90.3
	Yes	30	8.6
	Do not know	4	1.1
Medication type	Oral	226	64.6
	Insulin	69	19.7
	Both	53	15.1
	Nothing	2	0.6
Hypertension	No	205	58.6
	Yes	137	39.1
	Do not know	8	2.3
Dyslipidemia	No	200	57.1
	Yes	150	42.9
Smoking	No	241	68.9
	Yes	97	27.7
	Ex-smoker	12	3.4
Herbal or traditional medicine	No	259	74.0
	Yes	85	24.3
	Not specified	6	1.7
FBG	Mean, SD	169.42	40.45
	Range	98.00	310.00
HbA1c	Mean, SD	7.83	1.00
	Range	6.00	13.00
Diabetes Control	Optimal (≤7.0%)	86	24.6
	Suboptimal (7.1-8.0%)	133	38.0
	Poor (>8.0%)	131	37.4

Practice in diabetes self-care

**Figure 1:**
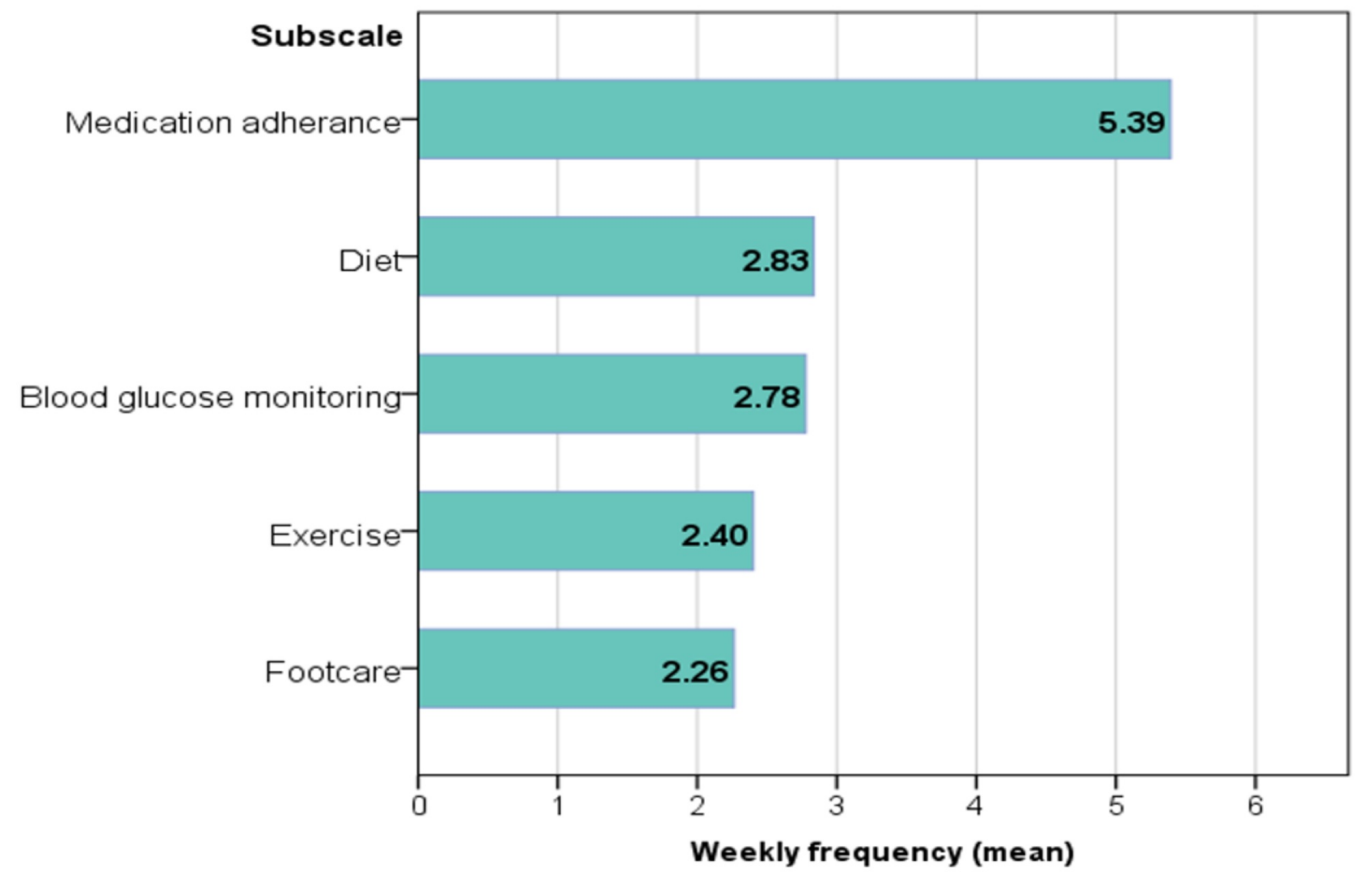
Practice in self-care activities. Bars represent the overall mean weekly frequency (days per week) of the given self-care activity subscale in the study population.

The SDSCA showed higher compliance with medication adherence (overall mean=5.39 days per week), followed by diet (2.83) and blood glucose monitoring (2.78), while footcare had the lowest level of practice (2.26) (Figure 1).

SDSCA scale internal consistency and SDCSA score characteristics

The internal consistency of the overall scale using the mean subscales (five items) showed Cronbach’s alpha=0.838. Inter-item correlation ranged between 0.349 and 0.634. Normality testing of SDSCA score distribution showed Kolmogorov-Smirnov (statistics=0.067; p=0.001) and Shapiro-Wilk (statistics=0.986; p=0.002), indicating that the variable is not normally distributed. Mean (SD) SDSCA score was 3.13 (1.13)/7. Median (P75) SDSCA score was 3.10 (4.00)/7 (these results are not presented in tables).

Correlation between the level of practice in self-care and glycemic control

**Figure 2:**
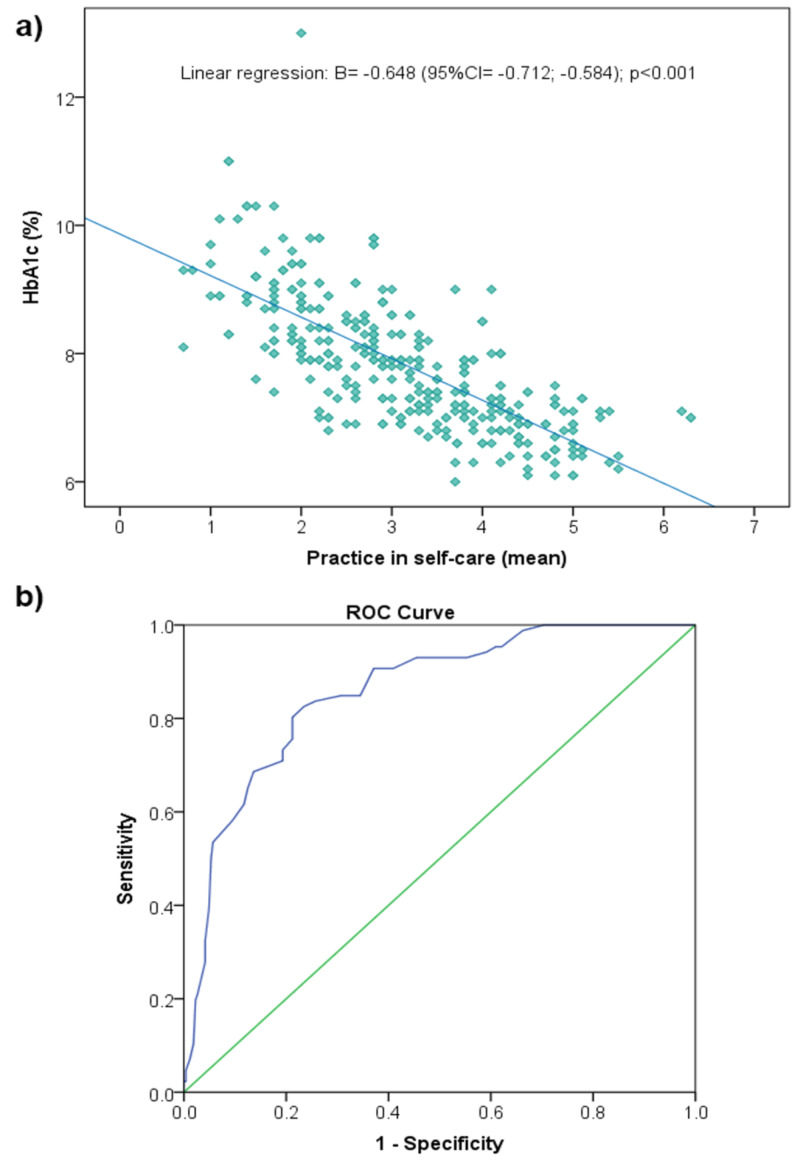
The correlation between self-care and the level of glycemic control. The upper panel (a) presents the linear regression of HbA1c level as a function of the SDSCA score, showing a significant negative relationship. The lower panel (b) shows the receiver operating characteristics (ROC) curve for optimal glucose control (HbA1c ≤7%) as a function of the levels of the SDSCA score; AUC=0.857 (95% CI=0.814, 0.901), SE=0.022. SDSCA: Summary of Diabetes Self-care Activities; HbA1c: hemoglobin A1c; AUC: area under the curve

The level of practice in self-care as expressed by the SDSCA score showed a negative correlation with the level of HbA1c, with a correlation coefficient R-squared=0.530 and regression coefficient B=-0.648 (p<0.001) (Figure 2A). By testing the significance of the SDSCA score in indicating optimal glucose control, defined as HbA1c≤7%, the ROC curve analysis showed AUC=0.857 (95% CI=0.814, 0.901), SE=0.022 (Figure 2B). Further analysis showed the best SDSCA score cutoff ≥3.5 (Youden’s index = 0.591) to indicate optimal glucose control with approximately 82.0% sensitivity and 77.0% specificity. Hence, this SDSCA score of ≥3.5 was used in the following analysis to indicate an adequate level of practice in diabetes self-management. According to this cutoff, 38.0% of the participants have adequate practice in self-management.

Sociodemographic factors associated with adequate practice in diabetes self-management

Participants aged >50 years had a lower percentage of adequate practice in diabetes self-management than those aged ≤50 years (26.2% versus 52.9%, p<0.001), respectively. With reference to single participants (54.9%), widowed (9.1%) and divorced (18.8%) participants had the lowest rates of adequate practice and 39.6% married participants had adequate practice (p<0.001). Regarding educational level, the adequacy rate was highest among highly educated participants (53.3%), followed by illiterate ones (41.7%), while it was lowest among the middle school level (18.4%) and the difference was statistically significant (p<0.001). Of note, accommodation ownership was associated with a lower level of practice (26.0%) as compared to rental (42.4%) (p<0.001), and freelancers and retired individuals had significantly lower levels of adequate practice in self-management as compared to the other professional categories (p<0.001) (Table 3).

**Table 3:** Sociodemographic factors associated with the level of practice in diabetes self-care. * Statistically significant results (p<0.05) SDSCA: Summary of Diabetes Self-care Activities

Parameter	Category	Practice level (SDSCA score)				p-value
		Inadequate (<3.5)		Adequate (≥3.5)		
		N	%	N	%	
Sector	North	40	57.1	30	42.9	
	West	37	52.9	33	47.1	
	East	44	63.9	26	37.1	
	Center	43	61.4	27	38.6	
	South	53	75.7	17	24.3	0.066
Gender	Male	109	61.6	68	38.4	
	Female	108	62.4	65	37.6	0.871
Age Category (years)	≤50	73	41.1	82	52.9	
	>50	144	73.8	51	26.2	<0.001*
Marital Status	Married	148	60.4	97	39.6	
	Single	23	45.1	28	54.9	
	Divorced	26	81.3	6	18.8	
	Widowed	20	90.9	2	9.1	<0.001*
Educational Level	Illiterate	7	58.3	5	41.7	
	Primary	22	73.3	8	26.7	
	Middle school	40	81.6	9	18.4	
	Secondary	85	68.5	39	31.5	
	University+	63	46.7	72	53.3	<0.001*
Nationality	Saudi	198	62.5	119	37.5	
	Non-Saudi	19	57.6	14	42.4	0.582
Accommodation	Ownership	111	74.0	39	26.0	
	Rental	103	53.6	89	46.4	<0.001*
Profession	Civil	57	46.0	67	54.0	
	Military	13	44.8	16	55.2	
	Freelance	52	81.3	12	18.8	
	Student	5	41.7	7	58.3	
	Retired	66	80.5	16	19.5	
	Unemployed	24	61.5	15	38.5	<0.001*
Household Monthly Income (SAR)	<5K	31	66.0	16	34.0	
	5-10k	74	56.9	56	43.1	
	10-15k	77	64.7	42	35.3	
	15-20k	25	59.5	17	40.5	
	>20k	10	83.3	2	16.7	0.339

Clinical factors associated with adequate practice in diabetes self-management

Lower levels of adequate practice in self-management were observed among participants with a longer duration of diabetes (<8 years) (adequate practice rate=19.2% versus 52.3%), retinopathy (18.1% versus 46.5%), cardiovascular complications (19.7% versus 41.9%), hypertension (28.5% versus 42.0%), and dyslipidemia (28.0% versus 45.5%) as compared to their counterparts, respectively, and all differences were statistically significant (p<0.05). Further, patients on insulin alone (23.2%) or insulin with OADs (28.3%) had lower levels of adequate practice as compared to those on OADs alone (44.7%), while the use of herbal and traditional medicine was associated with higher levels of practice as compared to non-use (55.3% versus 32.0%, respectively); both comparisons were statistically significant (p<0.05) (Table 4).

**Table 4:** Diabetes-related clinical factors associated with the level of self-care practice.

Parameter	Category	Level of self-care (score)				p-value
		Inadequate (<3.5)		Adequate (≥3.5)		
		N	%	N	%	
Diabetes duration	<8 years	95	47.7	104	52.3	
	≥8 years	122	80.8	29	19.2	<0.001*
Diabetes type	Type 1	17	63.0	10	37.0	
	Type 2	200	61.9	123	38.1	0.915
Cardiovascular complication	No	168	58.1	121	41.9	
	Yes	49	80.3	12	19.7	0.001
Retinopathy	No	131	53.5	114	46.5	
	Yes	86	81.9	19	18.1	<0.001*
Nephropathy	No	194	61.4	122	38.6	
	Yes	19	63.3	11	36.7	
	Do not know	4	100.0	0	0.0	0.283
Medication type	Oral	125	55.3	101	44.7	
	Insulin	53	76.8	16	23.2	
	Both	38	71.7	15	28.3	
	None	1	50.0	1	50.0	0.005*
Hypertension	No	119	58.0	86	42.0	
	Yes	98	71.5	39	28.5	
	Do not know	0	0.0	8	100.0	<0.001*
Dyslipidemia	No	109	54.5	91	45.5	
	Yes	108	72.0	42	28.0	0.001*
Smoking	No	152	63.1	89	36.9	
	Yes	59	60.8	38	39.2	
	Ex-smoker	6	50.0	6	50.0	0.635
Herbal or traditional medicine	No	176	68.0	83	32.0	
	Yes	38	44.7	47	55.3	
	Unknown	3	50.0	3	50.0	0.001*

Predictors for inadequate practice in diabetes self-management

Inadequate practice in diabetes self-management was independently associated with age >50 years (OR=2.00, p=0.043), rental accommodation (OR=0.42, p=0.04), freelance or independent job (OR=3.98, p=0.002), and longer duration of diabetes (OR=4.25, p=0.001) (Table 5).

**Table 5:** Independent factors associated with the level of self-care practice. Binary logistic regression, dependent variable (value): self-care practice level (SDSCA ≥3.5, median); * statistically significant result (p<0.05) SDSCA: Summary of Diabetes Self-care Activities

Predictor	Category	OR	95% CI		p-value
Age category	≤50 years	Ref	-	-	
	>50 years	2.00	1.02	3.89	0.043*
Marital status	Married	Ref	-	-	0.104
	Single	1.24	0.50	3.08	0.648
	Divorced	2.83	0.86	9.38	0.088
	Widowed	6.46	0.87	47.66	0.068
Educational level	Illiterate	Ref	-	-	0.004*
	Primary	0.64	0.11	3.72	0.621
	Middle school	2.62	0.47	14.59	0.270
	Secondary	1.37	0.29	6.55	0.690
	University+	0.50	0.10	2.38	0.381
Accommodation	Ownership	Ref	-	-	
	Rental	0.42	0.23	0.76	0.004*
Profession	Civil	Ref	-	-	0.019*
	Military	0.92	0.32	2.65	0.871
	Freelance	3.98	1.66	9.57	0.002*
	Student	0.38	0.08	1.95	0.247
	Retired	1.46	0.62	3.45	0.386
	Unemployed	1.72	0.68	4.35	0.249
Diabetes duration	<8 years	Ref	-	-	
	≥8 years	4.25	1.82	9.92	0.001*
Cardiovascular complication	No	Ref	-	-	
	Yes	1.06	0.42	2.67	0.902
Retinopathy	No	Ref	-	-	
	Yes	1.90	0.88	4.13	0.103
Medication type	Oral	Ref	-	-	0.644
	Insulin	0.98	0.37	2.61	0.967
	Both	0.57	0.22	1.44	0.235
		1.73	0.06	48.88	0.748
Hypertension	No	Ref	-	-	
	Yes	0.69	0.34	1.41	0.312
	Unknown	.00	0.00	.	0.999
Dyslipidemia	No	Ref	-	-	
	Yes	1.25	0.67	2.35	0.487
Herbal or traditional medicine	No	Ref	-	-	
	Yes	.41	0.05	3.12	0.386

## Discussion

Summary of findings

The present study explored the levels and determinants of practice in self-management among patients with type 1 and type 2 diabetes. These patients were registered and followed at the PHCCs in Jeddah City using a validated and frequently used tool. It showed unsatisfactory practice in self-management indicated by an overall SDSCA score=3.13/7 and below-cutoff scores observed in approximately two-thirds of the population, without much difference among the participating PHCCs. Lower levels of practice were observed in footcare and glucose monitoring. The level of practice was linearly correlated with the HbA1c level, and SDSCA ≥3.5 was associated with optimal glycemic control (HbA1c ≤7%) with 82.0% sensitivity and 77.0% specificity. Although several sociodemographic and clinical factors were associated with the level of practice in self-management, inadequate practice was independently predicted by older age (>50 years), independent job, and longer duration of diabetes, while accommodation rental was a protective factor. 

Low levels of glycemic control

Findings from the present study suggest that only one-quarter of patients with diabetes achieve optimal glycemic control, based on an HbA1c level ≤7%, with no difference between type 1 and type 2 diabetes. Data from local studies showed comparable findings. Two studies from Riyadh, in the central province of the Kingdom, showed a controlled diabetes rate as low as 18.5% and 21.1% among patients with type 2 diabetes based on HbA1c levels [1,7]. Another study from Tabuk city, in the northern province, showed 25.1% of satisfactory glycemic control among patients with type 2 diabetes attending a diabetic clinic; however, this study used fasting blood glucose (FBG) level and not HbA1c level [26]. Comparable percentages of controlled diabetes were reported in the eastern and southeastern provinces [27-28] while lower rates were reported in Al Madinah City [12]. Altogether, these data indicate an urgent need for national measures to improve diabetes control. On the other hand, there was a downhill linear correlation between the SDSCA score and the HbA1c level, signifying the importance of self-management in achieving optimal diabetes control.

Levels of practice in self-management

The present study used various methods to assess the level of practice in diabetes self-management based on SDSCA scores. Sub-scale scores showed relatively good practice scores in medication adherence (5.39 days per week), indicating that participants complied with their prescriptions for more than five days per week on an average. However, lower scores were observed for other self-care dimensions such as exercise, diet, glucose monitoring, and footcare. Analysis of the overall SDSCA score showed a low mean score (3.13) with 75% of the participants having score ≤4. Reports from other local studies showed comparable results. Al Johani et al. used the SDSCA scale and reported a similar trend in sub-scale scores. In this study, the medication adherence score was the highest (6.26 days per week) while very low scores (2.24-3.60) were observed for other dimensions such as exercise, diet, blood sugar checks, and footcare. The overall mean score (3.72) was slightly higher than that observed in our study [12]. Another study among patients with type 1 and type 2 diabetes conducted in Al Hada City, Makkah Province, used the SDSCA by converting the raw score into 0-100 scaled scores and showed similar trends, with medication adherence having the highest index of self-care (94.7%), followed by footcare (53.4%) and exercise and diet (approximately 41-42%); whereas blood glucose monitoring yielded the lowest score (22.4%) [14]. Further, to categorize the participants into adherent or non-adherent, we determined the cutoff value of the overall SDSCA score that is best associated with optimal glycemic control defined as HbA1c level ≤7% using ROC curve analysis. The model showed that SDSCA score reliably discriminated optimal from suboptimal glycemic control with AUC=0.857, and SDSCA ≥3.5 was associated with optimal glycemic control with 82.0% sensitivity and 77.0% specificity. According to this model, we estimated that only 38.0% of participants had adequate practice in self-management. This indicates the great gap in diabetes management in Jeddah PHCCs and highlights the need to intensify patient’s education for self-care to achieve substantial benefits in glycemic control.

Urgent need for interventions to promote self-management at the national level

Patient education for self-management is documented to be a crucial and cost-effective strategy to enhance practice in self-management and improve diabetes control [8]. This prompted physicians’ and researchers’ efforts to design various strategies to promote patient education for self-care [15-16]. A systematic review, including several studies from Saudi Arabia and the Gulf Cooperation Council (GCC) countries, studied the effect on glycemic control of various self-management interventions among patients with type 2 diabetes. Results showed that the majority of interventions induced a positive change in HbA1c levels [29]. Another systematic review showed that interventions to improve the patients’ knowledge, skills, and aptitude to perform self-management activities were highly effective and enabled a significant reduction in HbA1c levels. Additionally, the authors demonstrated that interventions were more effective when performed by a multi-disciplinary team and for a sufficient duration [10]. These interventions should be adapted to the population’s characteristics by targeting the most at-risk subgroups, which should be identified in each setting. In the present study, elderly patients and those with a longer duration of diabetes, as well as those having independent jobs, were identified as having the lowest levels of practice in self-management. Further, patient education interventions should include programs that improve patient’s health literacy, which was demonstrated to improve compliance in self-care activities [30]. Other strategies used technology-based solutions, such as the usage of smartphone applications and gamification, to alleviate further obstacles to self-management and produce promising results in the Kingdom [17-18].

Limitations

The present study did not explore patients’ participation and exposure in self-management education programs, which would enable determining further gaps in management and tailoring a more comprehensive intervention in the PHCC. Other factors, such as health insurance, were not explored, which may indirectly explain poor self-management among some subcategories of patients such as independent workers.

## Conclusions

Two-third of diabetes patients being followed at PHCCs have poor overall practice in self-management, especially in footcare and blood glucose monitoring. This is associated with suboptimal glycemic control among the majority of the patients, indicating the importance of self-management to improve diabetes control. Specifically, there is a crucial need to promote exercise, diet, and footcare, as these dimensions yielded the lowest levels of practice. Elderly patients, longer duration of diabetes (>8 years), and independent jobs were strong predictors of poor self-management practice, emphasizing the need for intensive intervention among these categories of patients. The patients' health literacy and education for self-management should be considered the standard of care for patients with diabetes in all PHCCs. In addition, the continuous adaptation of the content and monitoring of efficacy is essential. The use of novel technologies could be useful in alleviating obstacles to self-management.
